# Minimal Genome, Maximal Effect: How Polyomavirus Genomes Are Capable of Complex Pathogenesis

**DOI:** 10.3390/v18050533

**Published:** 2026-05-01

**Authors:** Michaela Lano, Barry Milavetz

**Affiliations:** Department of Biomedical Sciences, University of North Dakota School of Medicine and Health Sciences, Grand Forks, ND 58203, USA; michaela.lano@ndus.edu

**Keywords:** viral minimalism, polyomavirus, Simian Vacuolating Virus 40, genome economy

## Abstract

The Polyomaviridae family contains members known for achieving high seroprevalence within their target species despite a limited genomic economy. Minimalism, by definition, allows for the clarification and streamlining of purpose via the removal of unnecessary or distracting components. Among viruses, Simian Vacuolating Virus 40 (SV40) and other polyomaviruses are master minimalists, achieving efficient replication and persistence with compact genomes of approximately 5 kb in length. This review examines how polyomaviruses employ limited genetic material and simple structure to participate in complex functions and interactions, highlighting minimalism as both an evolutionary and functional advantage. Polyomaviruses make the most of their compact genomes in each stage of the viral lifecycle through the production of multifunctional early proteins and cis-regulatory elements, utilization of alternative splicing and host infrastructure, and organization of compact structural proteins. This allows for the successful replication and proliferation of virions while also reducing evolutionary pressure and promoting host immune evasion. Examination of the implications of polyomaviral minimalism illustrates that genome economy is not a constraint, but rather a driver of biological sophistication.

## 1. Introduction

**Figure 1:**
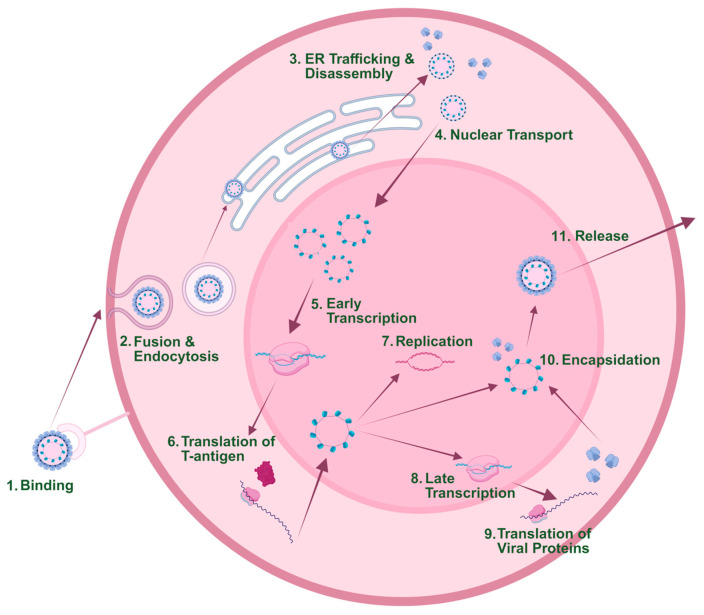
Lifecycle of SV40. Simian virus 40 (SV40) initiates infection by binding to the ganglioside GM1 receptor at the host cell surface, triggering caveolae-mediated endocytosis. Following internalization, virions traffic through endosomal compartments to the endoplasmic reticulum (ER). In the ER, partial disassembly of the viral capsid occurs. The partially disassembled viral particle is transported to the nucleus. There, the viral minichromosome is transcribed by host RNA polymerase II to produce early mRNAs encoding large T antigen and small t antigen. These proteins are translated in the cytoplasm and re-imported into the nucleus, where large T antigen initiates viral DNA replication. Viral DNA replication proceeds bidirectionally, producing a population of viral chromosomes. Then, late transcription of structural genes encoding VP1, VP2, and VP3 occurs. Capsid proteins are synthesized in the cytoplasm and imported into the nucleus, where encapsidation of newly replicated genomes occurs to form progeny virions. Progeny virions accumulate in the nucleus and are released upon host cell lysis. Created in BioRender. Lano, M. (2026) https://BioRender.com/ybk4nrh.

Simian Vacuolating Virus 40 (SV40) is a member of the polyomavirus family and is characterized by a highly compact genome of approximately 5 kb encoding only six proteins. This minimal genomic architecture is conserved across polyomaviruses, which generally exhibit similarly compact genome sizes. Despite this apparent limitation, polyomaviruses have achieved remarkable evolutionary and epidemiological success. Evolutionary studies have indicated that these viruses have achieved longevity, co-evolving alongside their hosts for roughly 500 million years [1]. In addition to their considerable longevity, Polyomaviruses are also notable for their high prevalence within their target host populations. Approximately 50–80% of humans are estimated to be seropositive for JC virus or BK virus [2,3,4]. Comparable rates of SV40 seropositivity are observed in captive nonhuman primates, including rhesus macaques and baboons [5,6]. These findings highlight the widespread and efficient transmission of polyomavirus family members across diverse host species. Rather than representing a constraint, the small genome of polyomaviruses delivers multiple functional advantages throughout the life cycle of the virus infection cycle. In the SV40 life cycle, SV40 chromosomes transported to the nucleus undergo a series of regulated steps that allow for the successful completion of early transcription, repression of early transcription, late transcription, replication, and encapsidation (Figure 1). Upon the completion of these processes, newly formed SV40 virions are released from the host cell. Genomes of SV40 and other polyomaviruses are composed of double-stranded DNA organized into chromatin that is remarkably similar structurally to that of the host cells that they infect. Therefore, polyomaviruses are adept exploiters of cellular regulatory factors and epigenetic regulatory pathways. The compact and uncomplicated genetic and structural organization by polyomaviruses allows for efficient viral replication, enabling these viruses to maximize biological output with minimal genetic material. In this sense, polyomaviruses are talented minimalists, able to achieve success through simplicity at each point in the viral life cycle.

## 2. Minimalism During the Viral Life Cycle

### 2.1. Transmission

Since the 1960s, the polyomavirus SV40 has emerged as a powerful model system for studying fundamental aspects of DNA virus biology, including replication, transcriptional regulation, and chromatin organization in a eukaryotic context. The discovery and most substantial route of transmission of SV40 in humans occurred following the delivery of millions of doses of contaminated polio vaccines [7]. However, this event does not reflect the true nature of transmission or tropism of SV40 and other polyomaviruses in their respective target hosts. SV40’s natural hosts consist of non-human primates, and it is not considered to be a true human polyomavirus. True human polyomaviruses are capable of transforming host cells and causing other severe pathology in immunocompromised individuals, and include Merkel cell polyomavirus, JC virus, and BK virus. Transmission of human polyomaviruses occurs during close contact, via respiratory or fecal-oral routes. Tropism of several human polyomaviruses is localized in kidney and urinary tissues, with viral shedding occurring via urine [8]. Polyomaviruses, including JC and BK viruses, are capable of establishing persistent or latent infections in various host cells and tissues, creating the opportunity to reactivate and shed virions in the future [8].

In addition to advantages conferred by latency, polyomaviral transmission benefits from compact viral genomes and simple virion structure. Minimalist structure allows for quick and efficient production of virions. The structurally simple, nonenveloped virions are well suited for persistence in both host tissues and the environment. The small, nonenveloped capsid confers resistance to environmental degradation factors such as heat [9], while the limited genome encodes a relatively small number of multifunctional proteins to maximize replication efficiency and immune evasion. These features allow polyomaviruses to leverage structural and genomic minimalism to maintain effective transmission cycles across various hosts and environmental contexts.

### 2.2. Early Transcription

**Figure 2:**
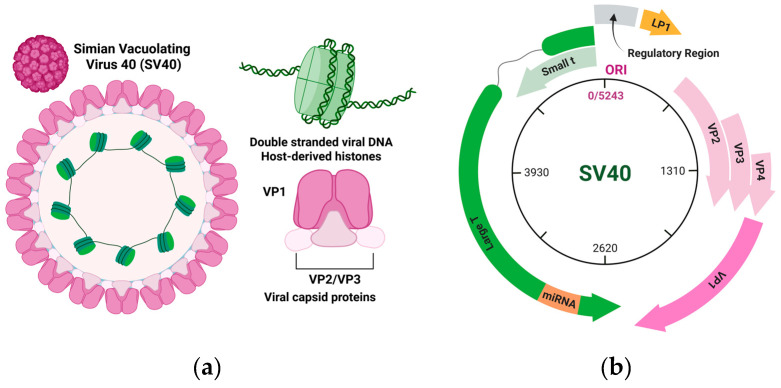
Structure and components of SV40 virions and genome. (**a**) The SV40 virion is a non-enveloped particle containing pentamers composed of viral proteins surrounding the viral genome. Each VP1 pentamer associates with minor capsid proteins, VP2 or VP3. Enclosed within the capsid is the circular double-stranded DNA genome, organized as a minichromosome through association with host-derived histones. Created in BioRender. Lano, M. (2026) https://BioRender.com/wsdy7lc. (**b**) The SV40 genome is organized into early and late transcriptional regions that are transcribed from a central regulatory region. The early region encodes the large T antigen and small t antigen via alternative splicing, while the late region encodes the structural proteins VP1, VP2, VP3, and VP4, as well as LP1 (agnoprotein). Created in BioRender. Lano, M. (2026) https://BioRender.com/7brgxl0.

The SV40 viral life cycle features early and late phases [10]. The early stage of SV40 infection starts with the binding of viral proteins to host cell ganglioside GM1 [11,12]. From there, virions are endocytosed to the endoplasmic reticulum [13,14,15], partially disassembled, and then trafficked further to the nucleus [16,17]. Upon entering the host cell nucleus, prior to genome replication, SV40 begins early transcription and translation of Large and Small T antigen (Figure 1). Early transcription in simian virus 40 (SV40) is initiated rapidly following nuclear entry of the viral minichromosome and is carried out by host RNA polymerase II in conjunction with cellular transcription factors. The early promoter resides within the noncoding regulatory region and contains GC-rich sequences, tandem 72 bp repeats, and binding sites for factors such as Sp1 and AP-1 that collectively drive transcriptional activation. This phase generates a primary transcript that is alternatively spliced to produce mRNAs encoding the large T antigen and small t antigen (Figure 2). Large T antigen is essential for viral replication, as it binds the origin of replication and orchestrates the recruitment of host replication machinery, while also modulating transcriptional control. Through interactions with key cell cycle regulators, including p53, large T antigen promotes host cell entry into S phase, thereby creating a cellular environment conducive to viral DNA synthesis [10].

In the context of viral minimalism, early transcription in SV40 demonstrates how a compact genome encodes multifunctional regulatory proteins that maximize limited coding capacity. The use of a single promoter and alternative splicing strategy enables the production of multiple functionally distinct proteins from a shared transcript. Moreover, the large T antigen plays multiple roles in DNA replication, transcriptional regulation, and host cell manipulation, effectively substituting for numerous factors that more complex viruses encode separately. The regulatory region itself is densely packed with overlapping control elements, allowing fine-tuned transcriptional responses without the need for extensive genomic space.

As infection progresses, early transcription is actively repressed to facilitate the transition to late gene expression. This repression is partially mediated by large T antigen itself, which binds to sites within the viral origin and early promoter region to inhibit further initiation by RNA polymerase II. This ensures a tightly coordinated temporal switch from early to late phases of the SV40 life cycle.

### 2.3. Replication

SV40 DNA replication is initiated at a well-defined origin within the viral regulatory region (Figure 2) and is driven almost entirely by host replication machinery. Following early gene expression, the large T antigen binds specifically to the origin of replication, inducing local DNA unwinding and the formation of a bidirectional replication fork [18,19]. Large T antigen also recruits and coordinates several host factors, including DNA polymerase α-primase, replication protein A, and topoisomerases, to establish a functional replisome [20]. Replication proceeds bidirectionally, producing genomes that are organized into chromatinized minichromosomes.

SV40 DNA replication highlights the virus’ reliance on a single multifunctional protein to orchestrate a complex, multistep process. The large T antigen not only recognizes the origin, but also functions as a helicase and recruiter of host replication factors, thereby eliminating the need for virally encoded replication enzymes. This strategy allows SV40 to maintain a compact genome while still achieving high levels of replication efficiency. Furthermore, the viral origin of replication is densely organized with overlapping binding sites that maximize regulatory control within a limited sequence space. By utilizing host enzymes and consolidating multiple activities into a single protein, SV40 exemplifies how polyomaviruses achieve replication competence with minimal genomic investment. As this strategy requires precise coordination between viral and host processes, particularly with respect to cell cycle regulation and the availability of replication factors, large T antigen has evolved to manipulate host signaling pathways to ensure that the cellular environment remains permissive for viral DNA replication. This integration of viral replication into host chromatin machinery demonstrates the efficiency of SV40’s minimal genome ability to achieve complexity in its interactions with the host cell.

### 2.4. Late Transcription

Late transcription in SV40 is activated following the onset of viral DNA replication and is driven by host RNA polymerase II acting on the late promoter within the regulatory region. This phase is characterized by a marked increase in transcriptional activity, producing abundant late mRNAs that encode the structural proteins VP1, VP2, and VP3 [10]. Initiation from the late promoter is heterogeneous, generating transcripts with multiple start sites that are subsequently processed through alternative splicing and polyadenylation. In addition, regulatory interactions involving the large T antigen and changes in promoter architecture contribute to the coordinated shift from early to late gene expression [10].

SV40 late transcription exemplifies how extensive protein diversity can be generated from a limited genomic template. The use of multiple transcription start sites, coupled with alternative splicing and differential polyadenylation, enables the production of several structural proteins from overlapping reading frames. This strategy minimizes genome size while maximizing coding potential, allowing SV40 to efficiently produce the components required for virion assembly. Moreover, the reliance on host transcriptional and RNA processing machinery reduces the need for virally encoded regulatory factors. The coupling of late transcription to DNA replication further streamlines the viral life cycle, ensuring that structural protein synthesis occurs only when sufficient genomes are available for packaging.

### 2.5. Encapsidation

Encapsidation in SV40 occurs within the host cell nucleus and involves the coordinated packaging of capsid proteins around newly replicated viral genomes. The major capsid protein VP1 forms stable pentameric units that constitute the outer shell of the virion, while the minor proteins VP2 and VP3 localize internally and contribute to genome stabilization and infectivity. Unlike many DNA viruses, SV40 packages its genome as a chromatinized minichromosome, with viral DNA remaining associated with host-derived histones throughout the assembly process (Figure 2) [10,21,22]. Capsid assembly is thought to proceed via the progressive association of VP1 pentamers with the viral nucleoprotein complex, rather than through the insertion of DNA into a preformed capsid. Specific and nonspecific interactions between capsid proteins and the viral genome, along with nuclear localization of structural components, ensure efficient and selective encapsidation [23].

In SV40 encapsidation, a limited set of structural proteins can mediate efficient virion assembly without the need for complex packaging machinery. The intrinsic ability of VP1 pentamers to self-assemble eliminates the requirement for dedicated scaffolding proteins or ATP-driven packaging motors. Additionally, the retention of host histones on the viral genome reduces the need for specialized viral DNA-condensing factors, effectively outsourcing genome organization to host-derived components. The minor capsid proteins VP2 and VP3 perform multiple roles, including linking the genome to the capsid interior and facilitating entry during subsequent infection. This integration of self-assembly, host factor utilization, and multifunctional protein design underscores the efficiency of SV40 encapsidation.

### 2.6. Vacuolization and Exit from Host Cell

SV40 appears to employ several unique mechanisms of viral release from host cells, not all of which are present in other polyomavirus family members. SV40 relies on a combination of VP4 viroporins, vacuolization, and stress-induced death pathways to release newly formed virions from host cells. A prominent mechanism of viral release, and namesake of the virus, is vacuolization. Vacuolization is a process that begins at the surface of the cell, when the viral VP1 protein binds to host GM1. This triggers a cellular signaling pathway involving Ras, Rac1, MKK4, and JNK. Viral particles are then trafficked to the Endoplasmic Reticulum, leading to increases in endocytosis, causing endosomes to accumulate and repeatedly fuse. These enlarged compartments then coalesce into numerous, giant vacuoles that fill the cytoplasm. The large number of vacuoles then leads to membrane insecurity in the host cell and therefore lysis [24,25,26].

The second process by which SV40 lyses host cells is through stress-induced death pathways. SV40 replication activities within a host cell cause numerous stresses to the host cell, including viral protein overproduction and DNA replication burden. The infection process can also damage or induce dysfunction in cellular organelles, especially in the mitochondria. This general damage can lead to induction of apoptosis signaling or lytic cell lysis by the host cell [27,28,29]. Other polyomaviruses such as JC, BK, and mouse polyomavirus also tend to cause apoptotic or necrotic cell lysis due to damage to host cell components [30,31]. This mechanism makes use of host cell signaling pathways, as SV40 and other polyomaviruses do not encode their own lysis machinery.

The last proposed, but not universally accepted, mechanism by which SV40 lyses host cells is viral protein VP4 acting as a viroporin [32,33]. SV40 VP4 is expressed late in infection and localizes to cellular membranes, including the endoplasmic reticulum, nuclear envelope, and plasma membrane. Its hydrophobic structure allows it to insert into lipid bilayers and likely form small pores or membrane defects. This increases membrane permeability, disrupting ion gradients and cellular homeostasis. VP4 is also thought to contribute to weakening intracellular membranes, aiding movement of virions and amplifying cellular stress alongside processes like vacuolization and cell death pathways. These effects lead to loss of membrane integrity, cell lysis, and release of newly formed virions. VP4 has been proposed to be the one truly encoded lytic factor in the SV40 genome, and other polyomaviruses do not seem to have analogous proteins [34]. It originates from the late coding region of the SV40 genome, a truncated product among a transcript where three alternate start sites encode VP2, VP3, and VP4 respectively. This example is one of many space-saving strategies used within the SV40 genome and structure to produce maximal biological output.

### 2.7. Host Immune Evasion

Once SV40 and other polyomaviruses encounter a new host, they employ an immune evasion strategy grounded in relying on limited immunogenicity rather than extensive immunomodulatory mechanisms. Unlike large DNA viruses that encode inflammatory modulators such as cytokine homologs, pattern-recognition receptor antagonists, or MHC downregulators, SV40 produces very few viral proteins and lacks dedicated immune evasion factors. However, this does not necessarily lead to reduced immune evasion. Rather, there are less antigenic viral proteins present, leading to weaker adaptive and innate immune responses. This contrasts with larger viruses, whose numerous foreign and immune evasion proteins can themselves become antigenic. SV40 further minimizes immune detection during entry by utilizing caveolar endocytosis and trafficking to the endoplasmic reticulum, where it partially unfolds and crosses the ER membrane via host ER-associated degradation (ERAD)-like machinery. This pathway avoids strong activation of Toll-like receptors, limits cytosolic exposure of viral DNA, and does not involve significant membrane disruption, thereby reducing the release of pathogen-associated molecular patterns and dampening innate immune sensing [17]. Additionally, SV40 replication occurs in the nucleus with relatively limited early cytopathic effects and without the production of highly inflammatory proteins, in contrast to viruses that induce cell lysis or robust interferon responses. In addition, SV40 does not substantially disrupt antigen presentation pathways, as it lacks dedicated mechanisms to downregulate MHC class I and does not markedly perturb endoplasmic reticulum homeostasis. This minimalist feature appears to extend to other members of the polyomavirus family. As their simple structure minimizes perturbation of host immune systems, polyomaviruses are able to largely evade host immune response and enter host cells without encoding sophisticated immunomodulatory components.

## 3. Minimalist Strategies

### 3.1. Chromatin Architecture

During infection, polyomaviruses organize viral DNA into a minichromosome with structure similar to host eukaryotic chromatin. Polyomaviruses utilize epigenetic regulation and chromatin architecture to regulate viral biological function and transition between active replication and latency [35]. Among genetically identical chromosomes within a host cell, populations of chromosomes with differing chromosome architecture are associated with various biological functions. In particular, histone modifications and nucleosome positioning have been found to guide viral functioning [21,22,36].

Dynamic histone modifications play a critical role in regulating SV40 transcriptional progression. Early in infection, enrichment of activating marks such as histone H3 and H4 acetylation promotes expression of viral early genes, whereas later stages are associated with redistribution of these marks and accumulation of repressive modifications over the early region, facilitating the transition to late gene expression. Data collected in previous studies supports the possibility that acetyl-H3 predicts future RNA polymerase II (RNAPII) positioning, acetyl-H3 and acetyl-H4 can be associated with active transcription, and H3K9me1 and H3K9me3 indicate repression of RNAPII binding [21]. SV40 does not encode its own chromatin-modifying enzymes, instead employing host epigenetic machinery to establish and remodel histone modifications. This reliance allows for control of viral gene expression to be achieved through modulation of chromatin state rather than the expansion of coding capacity.

Nucleosome positioning on the viral minichromosome is highly organized and plays a critical role in regulating access to the origin of replication and bidirectional promoters. A nucleosome-free region is maintained over the regulatory region, while precisely positioned nucleosomes flank this site and modulate accessibility to transcription factors and the viral replication machinery. Dynamic placement of these nucleosomes during the course of infection contributes to the switch between early and late gene expression [22,36]. These structural transitions are driven largely by host chromatin remodeling activities and the multifunctional viral Large T antigen, rather than dedicated viral factors. Strategic placement and remodeling of a small number of nucleosomes enables SV40 to regulate DNA accessibility and coordinate transcription and replication without expanding its coding capacity.

### 3.2. Alternative Splicing

SV40 utilizes alternative splicing as a key mechanism to maximize coding capacity within its compact genome and to temporally regulate gene expression during infection. The SV40 genome is organized into early and late transcriptional regions, each producing primary transcripts that undergo alternative splicing to generate multiple mRNA species from a limited number of coding sequences (Figure 2). Early in infection, the primary early transcript is alternatively spliced to produce large T antigen, small t antigen, and additional isoforms that play roles in viral replication, transcriptional regulation, and modulation of host cell cycle progression [10,37]. These proteins share common exons but differ in their exon combinations and reading frames due to differential splice site usage, enabling distinct functional domains to be encoded from overlapping regions of the genome.

During the late phase of infection, alternative splicing of late transcripts derived from a common precursor RNA yields mRNAs encoding the structural proteins VP1, VP2, and VP3, and VP4. VP2, VP3, and VP4 are produced from the same transcript but utilize alternative translation initiation sites rather than distinct splice variants, whereas VP1 is generated from a separate spliced mRNA [10,38]. The coordination between alternative splicing and alternative translation initiation ensures efficient production of capsid components while maintaining genomic economy. Regulation of splicing is influenced by host splicing machinery and viral regulatory elements that modulate splice site selection, contributing to the temporal switch from early to late gene expression.

### 3.3. Cis-Regulatory Elements

The SV40 genome features a highly compact cis-regulatory architecture that integrates multiple regulatory functions within a small noncoding regulatory region. This region contains the viral origin of replication, overlapping promoter elements, and numerous transcription factor binding sites arranged within a limited stretch of DNA [10,21]. Rather than encoding extensive regulatory proteins, SV40 relies on this densely packed cis-acting sequence organization to coordinate transcription, replication, and temporal control of gene expression using host and viral factors. The early promoter drives transcription of early genes immediately after infection, producing regulatory proteins such as large T antigen. Large T antigen then binds directly to one of two sites within the origin of replication within the same cis-regulatory region [39], leading to either initiation of transcription or repression early transcription within minimal genetic space.

The overlapping nature of functional elements within the regulatory region allows individual nucleotide sequences to participate in multiple roles, including promoter activity, transcription factor binding, and replication origin function. As SV40 infection progresses, the same compact regulatory region supports a transition from early to late gene expression, influenced by DNA replication status and changes in protein occupancy at shared cis-elements. This switch is achieved without expanding the genome, instead relying on dynamic interactions among a limited set of viral proteins, host factors, and multifunctional DNA elements. By embedding replication origins, promoter elements, and transcription factor binding sites within a single compact region, SV40 efficiently coordinates its life cycle while minimizing genetic content and maximizing functional output from a compact genome.

### 3.4. Large T Antigen

SV40 exemplifies viral minimalism through the multifunctionality of its limited protein repertoire, with Large T antigen serving as the central regulatory hub of the viral life cycle. Encoded by the early region of the genome, Large T antigen is a multifunctional protein that plays multiple roles in viral DNA replication, transcriptional regulation, and manipulation of host cellular pathways [20,40]. Its domain organization enables multiple functional activities, allowing SV40 to accomplish complex regulatory tasks without the need for additional specialized proteins.

A primary function of Large T antigen is the initiation of viral DNA replication. It binds specifically to the viral origin of replication within the noncoding control region and assembles into a helicase complex. This helicase activity unwinds the double-stranded DNA, recruiting host replication machinery, including DNA polymerase α-primase and other replication factors, to initiate bidirectional DNA synthesis. In this capacity, Large T antigen substitutes for multiple host replication factors by directly orchestrating origin recognition, DNA unwinding, and replication fork establishment.

In addition to its direct role in replication, Large T antigen functions as a transcriptional regulator. It modulates promoter activity, repressing early gene transcription once replication has commenced. This regulatory feedback ensures a temporal switch from early gene expression to genome amplification and subsequent late gene expression. Large T antigen also indirectly influences transcription by interacting with host transcription factors and chromatin-associated proteins, thereby altering the transcriptional landscape of the infected cell.

Large T antigen further contributes to viral replication by modulating the host cell cycle. It interacts with and inactivates key tumor suppressor proteins, including pRb family members and p53. These interactions drive the host cell into S phase, creating a cellular environment conducive to DNA synthesis and viral genome replication. Through these interactions, Large T antigen effectively reprograms the host cell cycle to favor viral propagation, illustrating its role as both a viral replication factor and a host regulatory effector.

Beyond these core functions, Large T antigen participates in additional protein–protein interactions that influence cellular signaling, DNA damage responses, and genomic stability. Its ability to engage multiple host pathways underscores its role as a multifunctional effector that coordinates viral replication with host cell manipulation. Collectively, the multifunctionality of Large T antigen allows SV40 to compress a wide array of biochemical activities into a single protein, reflecting a strategy of maximizing functional output while maintaining a compact genome.

### 3.5. Viral Proteins

Other SV40 proteins also illustrate viral minimalism by combining structural roles with additional functional activities that extend beyond their primary assignments. The capsid protein VP1 not only forms the structural shell of the virion but also mediates attachment to host cells through binding to the GM1 ganglioside receptor, linking structural assembly to the initial stages of infection and cell entry [24]. The minor capsid proteins VP2 and VP3, produced from the same genomic region via alternative translation initiation, assist in virion assembly and intracellular trafficking, and their hydrophobic properties enable interactions with cellular membranes that may facilitate steps leading to virion maturation and release. These proteins also contribute indirectly to late-stage infection by supporting proper capsid formation and potentially influencing membrane-associated processes. Multifunctional SV40 proteins perform multiple, sometimes overlapping roles. This strategy reduces genomic complexity while maintaining the ability to coordinate replication, assembly, and exit through efficient reuse of protein functions and host interactions.

## 4. Advantages and Constraints of Minimalism

SV40 illustrates how viral minimalism confers both evolutionary advantages and inherent constraints. By maintaining a small genome that encodes a limited set of multifunctional proteins, SV40 reduces the metabolic and temporal costs associated with genome replication and packaging, enabling efficient and rapid propagation. This compact organization favors evolutionary streamlining, where selective pressure eliminates redundant genetic material and preserves only those functions that are essential or that can be combined into multifunctional proteins. As a result, SV40 proteins such as Large T antigen can integrate multiple roles—ranging from replication initiation to transcriptional regulation and host cell cycle modulation—within a single molecule, maximizing functional output per unit of genetic information. This economy of coding capacity allows the virus to adapt to conserved cellular environments without needing to evolve an extensive repertoire of dedicated enzymes.

At the same time, viral minimalism imposes important constraints. Because SV40 depends heavily on host systems for replication and gene expression, its evolutionary success is reliant on the availability and compatibility of host factors. For example, the virus relies on host DNA replication enzymes such as DNA polymerases, as well as host RNA polymerase II for transcription and the spliceosome for RNA processing. These dependencies restrict SV40 to permissive cellular environments and limit its host range and tissue tropism. Minimal genomes also reduce redundancy, meaning that mutations affecting multifunctional proteins can have numerous downstream effects, constraining the evolutionary pathways available to the virus. In addition, reliance on host machinery exposes the virus to host defense mechanisms that can disrupt these interactions. Thus, while viral minimalism promotes efficiency and compactness, it also creates evolutionary trade-offs between functional versatility and robustness, shaping how SV40 interacts with and adapts to its host environment.

## 5. Conclusions

The life cycle SV40 and other polyomaviruses illustrate how a compact genome and structure can support a complete and coordinated infectious process. Throughout the process of viral replication, SV40 relies heavily on host cellular machinery while limiting its own coding requirements. A key feature of this strategy is the multifunctionality of the large T antigen, which contributes to transcriptional regulation, initiation of DNA replication, and alteration of the host cell cycle. In addition, the use of overlapping regulatory sequences, alternative splicing, and variable transcription start sites allows multiple functional products to be generated from a compact genome. Transitions between phases of the life cycle, such as the shift from early to late transcription and the relationship between replication and structural protein production, are closely linked. Encapsidation further reflects this economy, as VP1 self-assembly and the incorporation of host histones reduce the need for specialized viral components. Together, these features demonstrate how SV40 organizes its life cycle to maximize efficiency while maintaining a limited genomic repertoire.

## Data Availability

No new data was created.

## Abbreviation

The following abbreviation is used in this manuscript:

SV40	Simian Vacuolating Virus 40
